# Exploring the lived experience of performance-related health and wellbeing among flautists

**DOI:** 10.3389/fpsyg.2024.1401122

**Published:** 2024-10-23

**Authors:** Jiayi Wang, Graham F. Welch

**Affiliations:** Department of Culture, Communication and Media, IOE, UCL’s Faculty of Education and Society, University College London, London, United Kingdom

**Keywords:** performance-related health, flautists, lived experience, COVID-19 pandemic, river of experience

## Abstract

**Introduction:**

The study has investigated the lived experience of flautists, focusing on their experiences and perceptions of performance-related physical discomfort, injury and related mental health challenges that they might have encountered in practice and performance. The aims of the research have been to provide flautists with an opportunity to reflect on any physical or psychological performance issues in their own words, and to understand the subjective meaning of these experiences.

**Methods:**

A basic qualitative approach was used for gathering data. All the fieldwork was undertaken during or immediately after the Covid-19 pandemic. Consequently, the participating flautists were deliberately selected using professional networks on the basis of their answers to a pre-interview initial questionnaire related to the characteristics of their personal backgrounds and their experiences, as well as being able to participate remotely. There have been two data collection phases. Phase 1 was a specially designed pre-interview questionnaire drawing on themes from appropriate literature. Phase 2 built on the pre-interview questionnaire responses and was designed as semi-structured interviews, undertaken on Zoom, and included a ‘River of Flute-playing Experience’ activity during the interview. The ‘River of Experience’ method is an autobiographical research tool in which participants were asked to annotate key biographical experiences and challenges at various points along their drawing of a meandering river. The combined data analyses drew on thematic analysis.

**Results:**

The eight participants reported a personal history of performance-related health and wellbeing challenges that they had faced at some point in their lives. The findings reveal that flautists encounter health-related challenges associated with their practice and performances, and the data suggest that they each require an understanding of likely performance-related health challenges and supportive resources to assist them in managing these challenges.

**Discussion:**

The participants’ diverse experiences highlight the importance of managing health and the value of supportive social connections. They cope with health challenges by integrating passion, resilience, and adaptability into their lives, finding ways to grow and continue to thrive in their flute-playing journey. The study underscores the need for comprehensive health education and support resources for flautists, emphasising the significance of resilience and adaptability in fostering health, wellbeing, and success.

## 1 Introduction

Reports of musicians’ performance-related health and wellbeing issues have been increasing in recent years (Papageorgi and Kopiez, 2012). Musicians, particularly at an elite level, can experience health and wellbeing challenges in making and performing music as a direct outcome of the strenuous physical and psychological requirements of their profession (Zaza et al., 1998). For example, musicians often require long hours of practice and rehearsal, which may cause physical problems and even long-term damage (e.g., Llobet et al., 2007; Jabusch and Altenmüller, 2006). Such activities not only make physical demands, but also often need tremendous concentration, which can be mentally tiring. Practice, rehearsal and performance require the body to deal with the effort of playing, and the pressure of seeking to improve performing ability and quality (Llobet et al., 2007). Overall, such demands imply that professional musicians are required to have a comprehensive training in technique.

Professional musical activities have been associated with reports of general psychological ill-health in musicians (e.g., Kenny, 2011; Steemers et al., 2020). For example, Papageorgi and Kopiez (2012) discuss the ‘psychological and physiological demands of learning to play a musical instrument’ (p. 731). Because playing music puts high demands on the musculoskeletal and nervous system, personal competence in health maintenance needs to be taken seriously by all musicians. One of the most common psychological problems encountered by musicians is performance anxiety (e.g., Kenny, 2011; Mazzon et al., 2023).

Playing-related physical injury has been discovered to have a vital influence on psychological health (Ascenso et al., 2018) and has been linked with high levels of general psychological ill-health among musicians who perform at a professional level. It is also reported that the psychological features of musicians’ injuries often include an ignorance of the significant demands that playing makes on their bodies, and that an acceptance of pain is a part of music-making (Paull and Harrison, 1997). For example, a study of symphony orchestras in Denmark argued that ‘musicians reported higher emotional demands, lower job satisfaction, lower decision latitude, lower social support and lower sense of community’ (Ascenso et al., 2018, p. 3), and with a higher degree of perceived stress among female musicians.

However, in contrast, in considering the mapping of ‘core’ elements of happiness and wellbeing, Seligman (2011) theorised that musicians can score relatively highly on each of the five components of his 2002 PERMA model (Positive Emotion, Engagement, Relationships, Meaning and Accomplishment) and that this is higher than in the general population (Ascenso et al., 2018).

Despite the rapid rise of research in the field of performing arts medicine (*cf* Burwell, 2012; Sataloff et al., 2010), there is limited previous research that has examined flautists’ lived experiences of health and wellbeing issues (e.g., Fain, 2010; Ackermann et al., 2011; Lonsdale et al., 2014). It is unclear how flautists identify themselves as being injured, and how they might address such concerns. Consequently, the aims of the current research have been to provide individual professional flautists with an opportunity to reflect on any physical or psychological performance issues in their own words, and to understand the subjective meaning of these experiences for the participants. The research explores flautists who self-identify as healthy, as well as those who have experienced performance-related health issues in the past, and those who are currently facing such issues. It seeks to shed light on the challenges that flautists face within flute learning and performance.

A related aim of the study has been to examine the resources that flautists require and make use of in order to address any performance-related health issues and the challenges that are associated with practice, rehearsal and performance.

## 2 Methodology

### 2.1 Theoretical framework

Aaron Antonovsky came up with the concept of ‘*salutogenesis* – of the origins (*genesis*) of health (*saluto*)’ (Antonovsky, 1979, preface vii), which was derived from his efforts to research health rather than disease. One underlying question concerned the origins of health. During his investigations, he uncovered the following response: ‘The origins of health are to be found in a sense of coherence’ (Antonovsky, 1979, preface vii). For him, ‘A Sense of Coherence’ (SOC)

*‘…is a dispositional orientation that allows individuals to be more resilient to stressors in daily life, stay well and improve their health. It includes three components: comprehensibility, manageability and meaningfulness.’* (Antonovsky, 1993, p. 727).

Antonovsky’s salutogenic model refers to an approach that focuses on factors that promote human health and wellbeing, as opposed to factors that contribute to disease. His model demonstrates how personal, social, and environmental resources may be utilised to address difficulties and to contribute to the development of an individual’s health. His question and its answer together make up the basis for his *Salutogenic Model of Health* (SMH). The important conceptual elements within the SMH are related to stress, breakdown, resources, a Sense of Coherence (SOC), and health (Vinje et al., 2017). These are explained below.

Sense of Coherence (SOC) is the key concept of the salutogenic model, which involves comprehensibility (the stressors driven by an individual’s internal and external life environments are structured, predictable, and meaningful), manageability (the individual has access to the necessary resources to address the challenges presented by these stressors), and meaningfulness (these challenges require effort to overcome). A strong SOC supports individuals in perceiving life as structured, predictable, and meaningful in dealing with stress and maintaining health.

Antonovsky proposed that there is a need to understand what he termed as Generalised Resistance Resources (GRRs), with a focus on the experiencing of discomfort that is unrelated to diagnosis and disease (Vinje et al., 2017). He gave a clear explanation and definition of GRRs as ‘any characteristic of the person, the group, or the environment that can facilitate effective tension management’ (Antonovsky, 1979, p. 99). According to Antonovsky (1979), GRRs are resources that help individuals perceive lives as structured, predictable, and manageable to effectively deal with stressors. GRRs are categorised into three resources: ‘(1) adaptability on the physiological, biochemical, psychological, cultural, and social levels; (2) profound ties to concrete, immediate others; and (3) commitment of and institutionalised ties between the individual and the total community’ (Antonovsky, 1979, p. 100). Such resources may include the following elements: (1) material resources, such as money and food, (2) knowledge and intelligence—knowing the real world and acquiring skills, (3) ego identity—a sense of inner self that is integrated but flexible, (4) coping strategies, (5) social support, (6) commitment and cohesion with one’s cultural roots, (7) cultural stability, (8) ritualistic activities, (9) religion and philosophy (e.g., stable set of answers to life’s perplexities), (10) preventive health orientation, (11) genetic and constitutional GRRs, and (12) individuals’ state of mind (Horsburgh and Ferguson, 2012, p. 182; Idan et al., 2022, p. 93).

Life experiences and health behaviours are other factors contributing to salutogenesis. Salutogenesis is also facilitated by promoting overall health and wellbeing through engaging in positive health behaviours, including maintaining a regular exercise routine, a healthy diet, and the avoidance of dangerous habits such as smoking and excessive alcohol use.

The traditional medical view of homeostasis as the basic human condition—homeostasis being defined as a self-regulating process by which an organism can maintain internal stability while adjusting to changing external conditions (*cf* Billman, 2020)—was challenged in Antonovsky’s theory, where he introduces the fundamental philosophical view of ‘the human organism as prototypically being in the state of heterostatic disequilibrium as the heart of the salutogenic orientation’ (Antonovsky, 1987, p. 130). In Antonovsky’s book *Health, Stress and Coping* (Antonovsky, 1979), he suggested that disease, illness, and entropy (decline into disorder) are common occurrences rather than being the exception when it comes to otherwise self-regulated homeostatic processes that are occasionally disturbed, leading to pathology (Vinje et al., 2017). In this book, he emphasised the importance of specific resistance resources (SRRs), as he found them both numerous and frequently beneficial in specific circumstances of tension:

*‘They (SRRs) are many and are often useful in particular situations of tension. A certain drug, telephone lifelines of suicide-prevention agencies or an understanding look in the eyes of an audience to whom one is lecturing can be of great help in coping with particular stressors. But these are all too often matters of chance or luck, as well as being helpful only in particular situations.’* (Antonovsky, 1979, p. 99).

### 2.2 Research design and participants

The current research drew on Antonovsky’s theoretical framework of GRRs (Generalised Resistance Resources) and SRRs (Specific Resistance Resources) to make sense of flautists’ reported experiences of their health and wellbeing related to practice, rehearsal and performance. A basic qualitative approach was used for data gathering (Merriam and Tisdell, 2015). All the fieldwork was undertaken during or immediately after the Covid-19 pandemic. Consequently, the participating flautists were deliberately selected using professional networks as being able to participate remotely, as well as on the basis of the combined characteristics of their personal backgrounds and experience. The eight participants were all of a similar performance demographic, in that each had played the flute at an advanced level for some considerable time (on average 42 years).

Following an initial pilot study, the research involved two main phases of data collection. Phase 1 was a specially designed pre-interview questionnaire that drew on appropriate literature and also sought an initial commentary from a specialist medical consultant in performance-related injury and who is also a flautist. Phase 2 was built on the pre-interview questionnaire responses and was designed as individual semi-structured interviews, undertaken via Zoom.

The pre-interview questionnaire was designed to collect demographic and background information. It was completed by a diverse group of flautists, aged above 18 and with varying levels of experience, ranging from music students and amateur enthusiasts to professional flautists and experienced flute teachers. Participants included both male and female flautists from various geographical locations and cultural backgrounds. The first section of the pre-interview questionnaire collected demographic information, such as age, gender, level of education, and occupation, as well as details about their flute training and experience, including years of flute playing. The second section investigated any physical or mental health issues participants perceived to be related to flute playing. The final section inquired about the flautists’ stress levels before, during, and after the COVID-19 pandemic.

Using questionnaires as a preliminary data collection tool can significantly improve contextual understanding by collecting background information and tailoring interview questions to address specific aspects of the participants’ experiences. Patton (2002) emphasises that understanding participants’ backgrounds allows for more effective and targeted interview questions. In addition, integrating data through questionnaires can enrich results by providing a structured understanding of the participants’ context (Creswell and Plano Clark, 2018).

**Figure 1:**
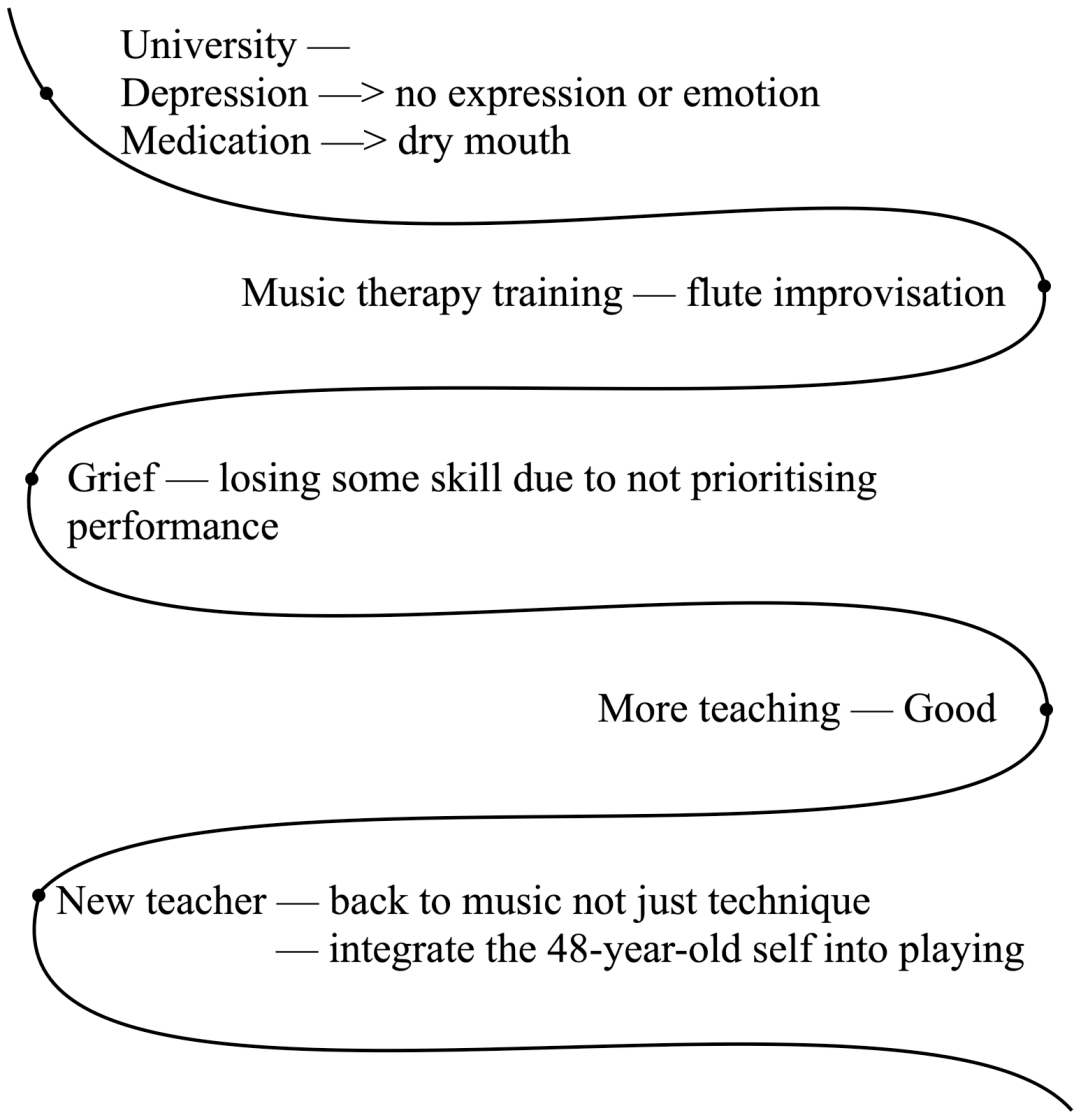
Participant Helen’s River of Flute-playing Experience.

**Figure 2:**
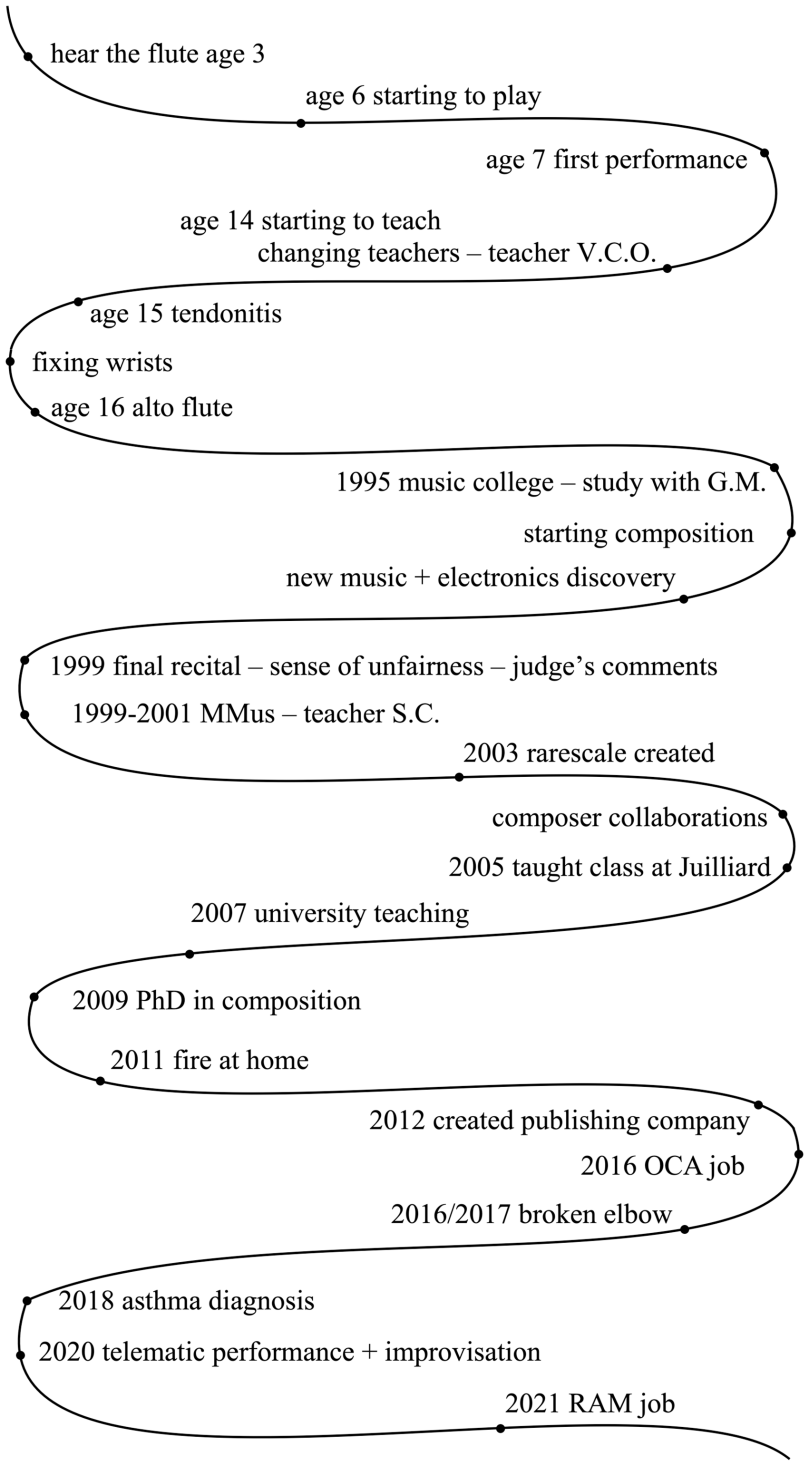
Participant Jane’s River of Flute-playing Experience.

The interview participants were selected based on their completion of the pre-interview questionnaire and their willingness to subsequently engage in a more in-depth interview. The aim of the interviews was to explore the lived experience of being a flautist (whether as a student, performer, teacher, or a combination of these roles) with health-related problems arising from flute playing, as well as their flute-playing history. The in-depth interviews were designed as ‘conversations with a purpose’ (Burgess, 1988) and were loosely structured around the participants’ reported difficulties. The interview also included an opportunity for participants to create a ‘River of Flute-playing Experience’ (*cf* Burnard, 2000; Denicolo and Pope, 1990; Taylor, 2011)—see examples from Helen and Jane (Figures 1, 2; names anonymised). Odena and Welch (2009) indicate that the aim of the River of Experience was to allow the participants to reflect on what had happened and what they experienced. This technique facilitated the structuring of an in-depth narrative that included salient features of the participant’s flute-playing history and was designed to generate opportunities for detailed reflection and discussion. Participants were asked to think back on their life experiences, write down their experiences and challenges at significant points of the river on paper, and subsequently discuss these with the researcher. The ‘River of Flute-playing Experience’ sheet also provided an opportunity for participants to reflect on their past musical lives (*cf* Kelly, 1955), which, for some participants, included the dual roles of being both a performer and a current flute teacher.

Interviews were fully transcribed and were analysed using specialist software NVivo (March 2018, version 12, Lumivero, *cf* Odena, 2007). Learning to play an instrument can be seen as a personal process that involves psychological, physiological, social, cultural, and aesthetic components. The themes for the interviews were related to the concepts of general and specific resistance resources (GRRs and SRRs), as well as physical, psychological and social challenges, and favoured coping strategies.

For example, in Figure 1, Helen’s River of Flute-playing Experience, she reported suffering from a dry mouth during her university studies. During the interview, she explained how she dealt with this problem by seeking support from her teacher. In this instance, the teacher could be seen as providing information that could serve as specific resistance resources (SRRs).

*‘I just had to always have a bottle of water next to me. And, in fact, my doctor wrote to the head of my music department and said that I would need to take sips of water while I was playing.’ (Participant Helen).*

The primary framework guiding the analysis was Antonovsky’s theory of salutogenesis, which emphasises factors that support health and wellbeing rather than those causing disease. This theory was applied to understand the factors contributing to flautists’ health and wellbeing, with a focus on enhancing their sense of coherence—a core component of Antonovsky’s theory, which includes comprehensibility, manageability, and meaningfulness. By organising the themes according to Antonovsky’s components, the research provided a more nuanced understanding of how various factors contribute to flautists’ health and wellbeing.

Throughout the analysis process, themes not directly related to Antonovsky’s theory were also identified and explored. This involved inductively coding the data to capture themes that naturally emerged from the participants’ experiences and integrating them into the interpretation. This iterative process of moving between inductive and deductive approaches ensured openness to all possible insights. The approach aligns with the qualitative research philosophy of being data-driven and responsive to the complexity of human experiences, ensuring that the findings are both theory-informed and grounded in actual data.

### 2.3 Research ethics

As the study involves human participants, prior to the commencement of data collection, a formal ethics application was reviewed and approved by University College London, reference Z6364106202010122, dated 30th October 2020. No children or young people identified as vulnerable were selected for this study. Potential participants were sent an information letter and confirmed their willingness to participate by email. A consent form was also provided, which made it clear that participation was voluntary. Permission to video-record the interview over Zoom was requested. Participants were also informed of their right to withdraw at any point, for any or no reason, in line with the British Educational Research Association (BERA) guidelines (2018). In addition, a contact email address for the researcher was also given so that participants could make contact, have any questions answered and report any concerns about the research process. Overall, all potential participants were informed of the focus and content of the study and signed a consent form prior to participation. To ensure that participants cannot be identified, they are given pseudonyms, which is the most prevalent method of anonymisation discussed in the literature (Clark, 2006; Saunders et al., 2015).

## 3 Results

In the application of Antonovsky’s theory, the following findings emerged:

### 3.1 Theme 1: General and specific resistance resources

Music could be seen as offering a General Resistance Resource for the participants since they felt that they could express themselves through playing the flute.

*‘So, if you play and have a certain level of technique but are also a 48-year-old woman with life history, that brings a different kind of maturity, I think, to my expression in my flute playing, which is a bit different; although, when I was at university, I was technically probably a lot better. I wasn’t a person who could express myself, whereas now I am a bit more.’ (Participant Helen, teacher and therapist).*

*‘Flute is, by and large, for me, a huge, positive mental health influence. One is it allows me these peak self-actualisation experiences when you are doing something really beautiful, which is a great antidote to ordinary stress and depression and feelings of loss and grief and sadness when you can do that. It’s the only thing that assuages such negative feelings. So that’s a huge plus.’ (Participant Albert).*

Positive relationships with students and working collaboratively with other flautists or flute teachers serve as examples of social interaction, and according to the Salutogenic Model of Health (SMH), such social support is crucial for promoting wellbeing.

*‘The other thing that happened was with [another professional flautist], and I decided to start these e-flute things. So, we did an e-flute festival first of all. And because everyone was at home, it was really successful. It was, you know, quite a big thing, [an] international thing. And when loads of international artists [were] there, and that was really a positive experience. I mean, I really enjoyed that.’ (Participant Chloe).*

*‘Now, as a teacher, you know, I try to get a feel of what the person I’m teaching is like, what they are learning…and have a really positive relationship with them. Because I think that’s just I know as a therapist that it’s within a positive, broader relationship that change can happen in my patients, and of course, learning the flute is about change, and that’s more likely to happen if I’ve got that really positive relationship with my students.’ (Participant Helen).*

*‘It’s all about having a relationship with somebody, and ideally working collaboratively with that person. So, as a result of my understanding of that as a teacher, I think my primary way of working is to have a positive relationship with my people. And working collaboratively with them towards whatever goals we set together.’ (Participant Helen).*

Participant Helen’s narratives indicate that she places a strong focus on the significance of building positive relationships with her students, which enhances their sense of being encouraged and motivated. Moreover, this approach enables her to identify and address any performance-related concerns at an earlier stage.

*‘I suppose in the last two years, I started having flute lessons for myself again. And that’s been a positive and a different phase of me kind of trying to integrate the skill I do have with the person I am now.’ (Participant Helen).*

*‘And so my career then developed into people started inviting me to play flute conventions. And so it developed into more of, kind of soloist. I started bringing lots of, because of my own kind of wellbeing journey. I felt like I’m in a really good position to help other people in their wellbeing.’ (Participant George, flautist, flute teacher and Feldenkrais method practitioner).*

Teachers could be viewed under the category of Specific Resistance Resources, as they provided specific support during the musical journey, such as the example reported above of Helen always having a bottle of water to compensate for her experience of a dry mouth.

*‘I did not trust my own judgment of how good I was or what my skill was. I really could not trust my own judgment, but I think it’s given me a clear idea of where my playing sets in terms of standard, because I’m getting feedback from somebody who I trust. I know [my new teacher] is always going to be nice. She’s not going to say anything really horrible, but she would not tell me something was great if it wasn’t, either. I have trusted her opinion for so long.’ (Participant Helen).*

Helen continues pursuing flute lessons and additional certificates, demonstrating her dedication to improving her skills for professional development and personal growth.

### 3.2 Theme 2: Physical challenges and adaptations

Flautists’ performance-related health challenges were explored. Physical and psychological health challenges included discomfort, like swelling and pain in the left forefinger, tinnitus, stress, chronic pain and chronic fatigue. These were examples of the challenges reported by the participants.

*‘Tendinitis was a big deal when I was a teenager. So, it was a long time ago. And it came about because I had a teacher who did not understand posture properly… He wasn’t a terrible teacher, but he had a quite old-fashioned view of posture. I was studying with him from the ages of seven to about thirteen–fourteen, and it caused a lot of damage physically. So, I used to have pains all the way up both arms, to the extent that I could not play… Firstly, I [needed to have] the right support in order to fix it. So, by that time, I had changed to a new teacher. So, I was changing my posture, and that helped. I went to an osteopath who really helped me to understand what my body was doing, how things worked, and how things were connected. And what he explained to me was that all of these problems [that] were raised there came from the top of my back.’ (Participant Jane).*

*‘It was just the swelling in my finger, which meant I could not bend it at all, and a swollen joint.’ (Participant Helen).*

*‘There was certainly a phase when I was a teenager, and I was playing in a lot of sort of youth groups, … when I would hyperventilate when I was playing, because I think the combination of anxiety making your breathing a bit erratic, with the fact that you are imposing erratic breathing because you are trying to play the flute which caused me to hyperventilate. And then that was really embarrassing, because I might have to leave the rehearsal because I needed to cover it, and everyone would see me. And then, being a teenager, expecting that to happen, almost made it happen. I made it worse because I was worried about being embarrassed at the anxiety showing.’ (Participant Helen).*

*‘Shoulder feels a bit odd…[because of] putting too much pressure on the keys… I’m pretty much aware of those… People have physical issues by holding the flute wrong, or they just do it for too long a time.’ (Participant Ruth).*

*‘It was in between my shoulder blades under my left shoulder… it was the chronic pain, was really, really that. It would just get very, very uncomfortable, kind of neck and shoulders… my neck and shoulders were just so tense… Every time I go on a holiday, I would have a massage to try and fix it.’ (Participant George).*

*‘I’ve got quite small hands and not much extension. And I was advised to play an open-hole flute, so I got an open-hole flute. And I learned to play the open-hole flute for quite a number of decades, and then suddenly about, I do not know, trying to think what age I was, I suppose, in my 50s, I mean, I’d been playing a long, long, long time, I suddenly got a problem with my right-hand thumb. And it was a sort of tendonitis, and I had an injection, and I had to put it in a splint. And then my wrist got locked, and I had to do so much exercise to get it all moving again.’ (Participant Olivia).*

### 3.3 Theme 3: Perceived psychological impact and emotional resilience

Amidst the experienced adversities, participants learned from challenges, found new ways to thrive despite the limitations imposed by the COVID-19 pandemic, and exhibited remarkable resilience. Their personal growth and enhanced ability to navigate challenges fostered a sense of adaptability to change.

*‘My shoulders were very tense, and I did a lesson [Feldenkrais lesson] where my arm came comfortably on the floor… I just immediately knew how this was my pathway towards myself, really… It [Feldenkrais] teaches you strategies to be more resilient. So, you become better able to regulate yourself during times of stress or anxiety. You suddenly have tools that you can use, and you have the awareness of some people’s problems playing… Feldenkrais teaches you to be more sensitive to the earlier points of going [awareness of the beginnings of discomfort]. This could get a bit uncomfortable, or I need a rest right now.’ (Participant George).*

Participant George reflects on a method of somatic education, the Feldenkrais method, that provided tools for resilience. It helped him become more aware of his body and its signals, allowing him to better manage stress and anxiety. His increased awareness and ability to regulate contributed significantly to his psychological resilience.

*‘PANDEMIC [sic]—extra challenge! Back to the loop of not touching the instrument and practice. I ended up practising piano more at home—a digital piano with headphones. Playing the flute at home alone wasn’t much fun, and I had to deal with noise issues—all the family members were at home, and everyone was working from home. I also lost lots of opportunities to explore the flute-playing scene in London.’ (Participant Ruth).*

Participant Ruth’s experience underscores the necessity of adaptability and persistence in order to maintain her musical practice during the pandemic. The limitations and challenges, such as noise issues and lost opportunities, had a negative psychological impact on her, as playing the flute at home alone was not much fun.

*‘In 2011, there was a fire in my house, and all my instruments were destroyed. My entire house was destroyed. So, it wasn’t a physical thing, but it was definitely a mental thing in that I had to deal with everything being lost… It was also just before I was going to submit my PhD. And I lost my entire PhD. And I had to start it again from scratch. I mean, literally, it was three months before submission… And at the point where I submitted it. I did not even have the instruments. I had to do it without any instruments in the end. Because they were destroyed. That was a challenge. But that was kind of interesting because I think resilience is important… I feel like every time something was problematic in my life, it gave me a new way forward. That was a very tough time. But it gave me an opportunity to again change everything if I wanted to change everything. So, I moved somewhere else. I got new instruments. I think it was very healthy in terms of my relationship with the flute. Because when the fire happened, I was just like, well, I’m not even a musician anymore. I have not got any instruments. How can I be a flute player if I do not have a flute? And I had to [be a flautist]… There are things the way I look at it is, there are things that we have control over, and they are the things that you can use in a positive way to question and learn. And then there are things that happen to us, like my house fire. It was an arson attack. Somebody did it. I had no control over that. There was nothing I could have done. So, I had to accept it, and I had to find a way of learning from it. And so that’s resilience.’ (Participant Jane).*

Participant Jane’s account of losing everything in her house fire, including her PhD work and instruments, highlights profound emotional resilience. After facing such a devastating loss, she managed to rebuild her life and develop a more positive connection with her music. Her story highlights how resilience involves accepting uncontrollable events and using those circumstances as opportunities for personal growth and change.

*‘Although these kinds of physical problems are seen as a negative, I think, actually for me, every time I’ve had some form of adversity, it’s been one of the most positive things that could have happened once I got through it. So, you know that experience, what happened was I managed to get back into, you know, great physical health. I changed all my posture when I was playing, and it removed all of the problems.’ (Participant Jane).*

Participant Jane regards physical adversity as a catalyst for positive change. She changed her flute-playing posture to overcome the physical challenges, which ultimately eliminated all the problems and improved her physical health. This experience exemplifies her resilience and ability to transform negative experiences into opportunities for personal development and progress.

*‘It wasn’t much of a challenge, but in society, we do not really connect with other human beings in a very meaningful or deep way. We, you know, might have relationships and partners, but [to experience] touch itself, we lack that experience. And when I started doing the training, suddenly being touched in a gentle and kind way by so many people, and was just so new for me… So, there’s something about accepting that connection. You know, because sometimes we are protective and that to do something about kind of accepting that, and also being able to connect with other people.’ (Participant George).*

Participant George also reflects on the importance of human connection. His Feldenkrais method training helped him connect with others. This illustrates how he found a way to build meaningful relationships, which in turn improves emotional resilience and fosters a strong sense of community.

*‘Because I’m not very good at technology. And now I’ve got a good setup here, I bought sort of proper equipment. But to start with [COVID-19 pandemic], I was using a small laptop, and I found it so tiring. But once I got used to it, and I got better equipment, it was alright.’ (Participant Olivia).*

Participant Olivia’s effort and eventual adjustment to technology during the pandemic demonstrates her resilience. At the beginning of the pandemic, her lack of technological skills and inadequate equipment were exhausting. However, she overcame these obstacles by acquiring better equipment and becoming accustomed to its use, which enabled her to adapt to the new conditions.

Participants’ quotes reflect their psychological resilience and adaptability in the face of adversity. From leveraging somatic education for stress regulation to overcoming significant personal losses, these narratives highlight how individuals can find strength and growth through challenging experiences.

### 3.4 Theme 4: Coping mechanisms and strategies—sense of community and support

Perceptions of stressors are personal and based on the situation (Antonovsky, 1979). Resources on a personal level are about understanding the lived experience of flautists’ challenges, utilising coping strategies, and maintaining motivation.

*‘I think there’s dealing with it when you are not feeling anxious, to try and reduce the overall level of anxiety and stress in life. And my advice would be practising things such as mindfulness and breathing, mindful breathing exercises. So, I would recommend specific exercises like square breathing, where you, you know, inhale for four, hold for four, exhale for four, hold for four. I know that, um, I sometimes get pupils or people who are stressed to touch their, feel their pulse in their neck, and then, if you breathe in, say, for four, and then breathe out for seven. What happens is, you can actually feel that as we exhale, our pulse slows down, and then it speeds up again. And once you could actually feel that, then you can understand the value in exhaling for a longer time to just reduce the overall levels of stress.’ (Participant Helen).*

**Figure 3:**
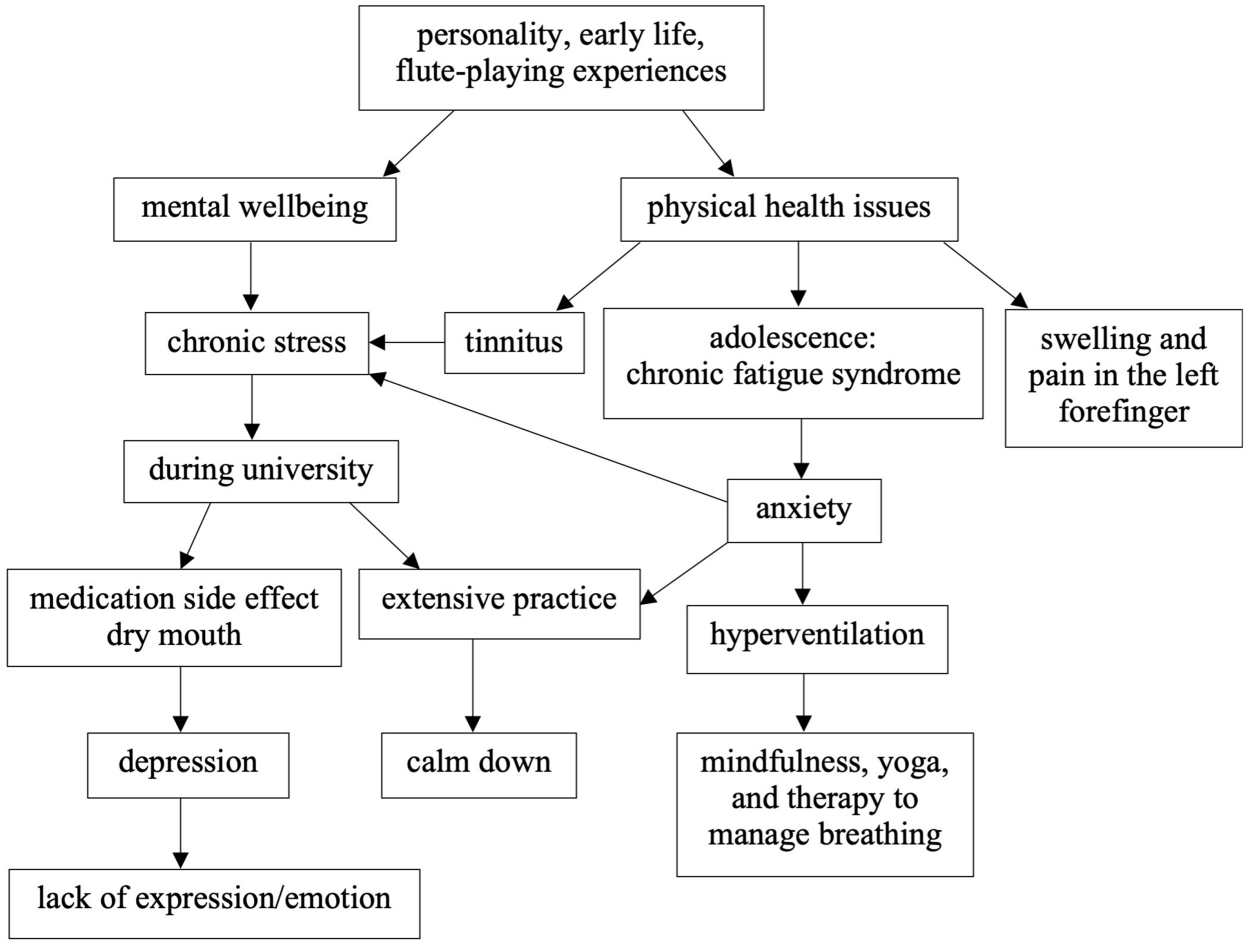
A model of Helen’s flute-wellbeing.

As depicted in Figure 3, participant Helen faced mental health issues, including chronic stress, depression, and anxiety. She used strategies to help reduce her overall stress levels, such as practising mindfulness, yoga, and mindful breathing exercises. The breathing techniques slow and deepen her breath to control hyperventilation.

*‘For making sure that I’m staying well and things like that, I mean, I’m not really into this, but I’m trying to do it so that I do not play without stopping for more than probably half an hour, maximum, before I put the flute down. You know, I would not want to just keep playing, but partly that’s because I’m starting to feel some pain as well. That’s the sign I need to do some stretching.’ (Participant Chloe).*

Participant Chloe manages physical discomfort and prevents injury by recognising and responding to her body’s signals. She avoids prolonged flute playing by taking breaks to prevent pain and uses any emerging discomfort as a cue to stretch.

*‘I found things like basic breathing exercises [to be] very good for anxiety. I do a little bit of yoga now. I had not really incorporated exercise into my routine very much as a young person… Stress and anxiety are, you know, [managed through] some physical exercise, looking after your body, and thinking about when you are an adult, and you have more control over your diet, and paying attention to what makes you feel good and feel balanced and healthy. So yeah, all of these lifestyle kind of elements, I think, have come into my thinking now about dealing with anxiety and stress.’ (Participant Sophie).*

Participant Sophie shared her experience of incorporating breathing exercises and yoga into her routine for managing anxiety. She highlights the importance of physical exercise, a healthy diet, and lifestyle changes in achieving balance and health.

*‘I decided during the pandemic to help people and make myself useful. So, I did, I did a lot of video recordings, practising live on Facebook. I did a lot of practice myself… I was being there for everyone else, but nobody was there for me, really. So, it came to a point. So, I was teaching, and I found teaching younger students on Zoom really stressful—parents guessing what time the lesson is… And I really went through a period of very, very, really intense anxiety. Actually, it just triggered a huge amount in me, and I went back to my old therapist and started working with him because everything was just too much, and it was, that was on top of the worry and stress about the pandemic itself. And the positive that came out with that is I learned a lot about how I deal with stress and anxiety. And I learned how to best manage it so that there’s been a huge positive impact from it, actually. But it was difficult. And when I came out of the pandemic, I got more teaching in the school that I taught, and I changed where I teach my Feldenkrais lessons from a huge positive impact was teaching Feldenkrais online classes. I had loads of people coming to my classes and did loads of workshops. So, overall, the pandemic helped me to learn a lot about myself, and I’ve grown.’ (Participant George).*

During the pandemic, participant George supported others by conducting online sessions, but this led to his own stress and anxiety. He sought help from his therapist and learned to manage these issues. Although he experienced challenges, he found a positive impact through teaching Feldenkrais lessons online, including enhanced self-awareness and personal growth.

Resources on a relational and social level are about understanding the importance of social support, such as from other flautists and flute teachers and through the experience of flute performance, as well as the development of positive relationships between themselves (as teachers) and their students through the teaching and learning process. Flute teachers can have a strong sense of coherence due to strong social relationships and support. These participating flautists reported leveraging online platforms for peer support. A connection with fellow musicians and opportunities for collaboration were reported to foster a sense of community and shared experience.

*‘At that time [of the fire] there was a lot of support from the community. They did a lot of fundraising to help me buy a flute, and this kind of thing.’ (Participant Jane).*

*‘Sometimes because music is the way we express, I think sometimes that is where the stresses can come out as well, and maybe we hold physical tension. So, I mean, in terms of that, I know lots of people who have had issues in different kinds of ways, some of them physical, some of them mental, some of them performance anxiety. I mean, I’ve taught students with performance anxiety that’s so severe that they cannot even approach a performance without crying. And I feel like there’s a way of overcoming all of those things, [this is] a lot of my work with the performance students. We have an online performance class once a month. And when they first come, some of them are really nervous. A lot of my job is to help them understand that it’s okay, and it’s normal. And that nervousness and excitement are similar things.’ (Participant Jane).*

*‘Meeting lots of other people who are interested in the flute, even the ensemble playing, not so much the playing, but just being around other people, and having good social networks and connections, and making new friends. Those are all things that are extremely important for mental health.’ (Participant Albert).*

*‘Having done that PhD, and having thought more about the way that we set up the rules, I suppose, of flute playing, or of music making for students, and have thought more about the social relations between a teacher and a student and the environment that we sort of set up, and this idea of power… which was something I had not really thought about when I was teaching before.’ (Participant Sophie).*

*‘I reflect back on myself as a student, wanting to model or wanting to use my teachers as models for my playing and for the kind of musician I would want to be. And I think for a young person who works one-to-one with a teacher. It’s a very powerful position to be in, you know, when you are that teacher, because you are a sort of a gatekeeper to opportunities. And if that student is serious about making music part of their life, then you have quite a heavy responsibility as an educator.’ (Participant Sophie).*

*‘At the beginning of the pandemic, I decided to make myself useful, so I created Flute Online… We have people from all over the world, and they join me, and they practise along with me… So, I decided during the pandemic to help people and make myself useful.’ (Participant George).*

Participants discuss a variety of strategies to manage physical and mental health, including taking breaks, incorporating physical exercise, yoga and breathing techniques, and seeking help from a therapist. Their experiences reflect journeys towards personal growth and adaptation to challenges, especially during the pandemic.

## 4 Discussion

Hours of daily training are required for expert musical performances, yet the physical and mental load and fatigue of playing have been recognised as risk factors for performance-related pain and injury. There are other causes of fatigue, not directly arising from the affected muscles, such as ‘the general physical aspects of your body (insufficient sleep, failing to adopt a balanced diet, a sedentary lifestyle, and the existence of concurrent disease)’ (Llobet et al., 2007, p. 3), and also mental fatigue (related to stress, mental overload, anxiety, perfectionism, constraint, choking under pressure or interference from other activities; e.g., Ascenso et al., 2018; Llobet et al., 2007). In conclusion, fatigue is multi-faceted and can accumulate and increase the risk of performance-related injury or illness. High-performing musicians are seen as motor athletes (Dick et al., 2013; Mazzon et al., 2023). A majority of student and professional musicians have been reported to suffer from one or more performance-related health issues (e.g., pain, overuse syndrome, tendonitis) over the course of their music journeys (McCrary and Altenmüller, 2020).

Although Antonovsky’s theory provided a useful lens for understanding the data, another significant aspect discovered beyond the specific scope of salutogenesis was the ergonomics and instrument design, particularly the weight and design of the flute. For instance, participant Olivia plays the open-hole flute. Over several decades, the challenges of holding the open-hole flute led to physical problems, specifically tendonitis in her right-hand thumb. This condition prevented her from keeping her thumb in a natural position, making it difficult to cover the holes.

In addition, flautists’ performance-related health problems concerning physical injuries can be typically depicted as overuse syndromes, such as tendinitis and tenosynovitis,^1^ and which—in mental health terms—can be described as neurological impairments (e.g., Guptill, 2011; Jankovic and Ashoori, 2008). Participant Jane also experienced tendonitis when she was at a young age, as shown in Figure 2. The impact of physical and mental health problems can result in discomfort, illness, and injury in professional flautists, which can inhibit artistic development. Approaches to healthy practising can also be derived from experienced music teachers’ recommendations, as well as from encountering the biographies of historical musicians. For example, in Clara Wieck-Schumann’s opinion, as a child instrument learner, she was not encouraged to practise more than three hours per day in order to avoid physical and mental exhaustion (reported by Papageorgi and Kopiez, 2012). Altenmüller and Kopiez (2010) propose the significant importance of having general physical endurance, as well as regular physical exercises of the hands, such as finger stretching—based on the view of a piano teacher, Friedrich Wieck (Wieck, 2005). Therefore, the acquisition, development and application of reflective powers are required in instrumental teaching and learning. Instrumental teachers need to develop constantly through their ongoing experience and reflection to support students who have questions.

The 48-year-old flautist Helen has held roles as a flute teacher and music therapist throughout her career. Figure 3 shows the results from the interview and her River of Flute-playing Experience, illustrating that Helen has suffered from tinnitus, chronic stress, chronic fatigue syndrome, and swelling and pain in her left forefinger. These challenges have impacted her professional and personal life. She copes with these issues by focusing on her own routine of practice, yoga, mindfulness, therapy, and specific techniques. She seeks medical advice and therapy when needed.

Helen suffered from chronic fatigue when she was a teenager. The fatigue was not caused by flute playing and is considered a pre-existing condition. However, playing the flute can worsen it and cause her anxiety. She faced challenges, including hyperventilation triggered by anxiety, which made her embarrassed during rehearsals. To manage her breathing, she practised breathing exercises described in Theme 4.

Helen experienced significant depression during her time at university. The medication she took caused dry mouth, and her depression severely impacted her flute performance. She also reflected that she played the piccolo a lot at university, which led to slight hearing damage. Furthermore, her tinnitus became permanent a few years ago, which increased her stress levels. To reduce anxiety and improve her overall wellbeing, she adopted various strategies such as extensive practice to calm down and integrating yoga and mindfulness into her life.

These ongoing challenges have an impact on her stress levels and flute-playing abilities, highlighting the necessity of adapting to the challenges. She uses specific techniques to manage her chronic fatigue and stress, improve her physical health, and promote mental wellbeing. While these performance-related issues are significant challenges for Helen, she also fosters resilience and adaptability throughout her journey.

Furthermore, Helen places a strong emphasis on building positive relationships with her students, which enhances their sense of encouragement and motivation. This approach also enables her to identify and address any performance-related issues at an earlier stage. Additionally, she continues to take flute lessons and pursue additional certificates, demonstrating her commitment to improving her skills for both professional development and personal growth, which shows her not to be held back by her health issues.

In summary, Helen copes with long-term performance-related health and wellbeing issues while adapting to the effects of her health conditions on her flute playing. Her experiences exemplify her extraordinary resilience and adaptability when confronted with health challenges. Her dedication to flute teaching and passion for music, along with her ability to build positive relationships that promote wellbeing, make her a dedicated flute teacher.

**Figure 4:**
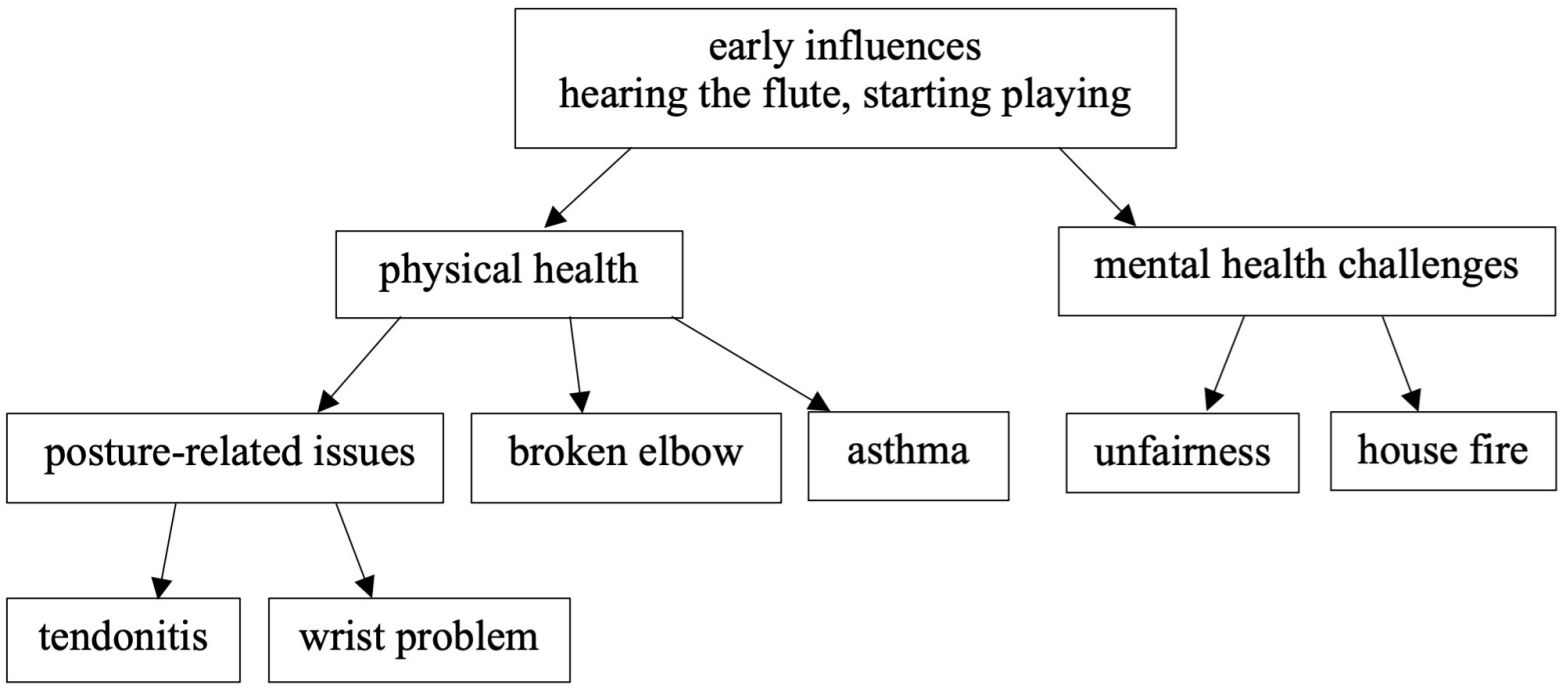
A model of Jane’s flute-wellbeing.

As depicted in Figure 4, the flute-wellbeing model of 46-year-old participant Jane, based on her interview and the River of Flute-playing Experience data, outlines the contributing factors to understanding performance-related health and wellbeing issues.

Jane’s first positive experience with the flute occurred when her parents took her to a concert at the age of three, and she expressed her desire to play the flute on stage. She began playing the flute at six. The concept of ‘challenge’ is drawn from Jane’s interview, in which she believes that challenge has kept her playing the flute for so long, as it pushes her to continuously improve. She stated, ‘There’s always something else to learn, always something else to explore, always another piece of music to discover, another musical culture to discover… I’m doing more and more improvisation-based work. I’ve always done a lot of collaborations, and those collaborations kind of spark ideas off each other.’ For Jane, challenge serves as a resource, helping her practise, improvise, collaborate, and find inspiration.

When Jane was a teenager, she had tendonitis due to hypermobility and postural issues. This issue arose because she had a flute teacher who did not understand posture properly. Jane began learning the flute at the age of seven. Her first flute teacher was brilliant, but then, when she was seven, she changed teacher and started studying with someone else who did his best but had a quite old-fashioned view of posture. Jane studied with him from the age of seven until around fourteen, which caused significant physical damage. However, she later adjusted her posture, which helped alleviate her problems. She also consulted an osteopath who helped her understand what her body was doing, how things worked, and how things were connected. Changing her posture when she was playing removed all her issues.

Jane also experienced wrist problems due to improper playing posture and hand positions, as instructed by a flute teacher who insisted on a rigid posture that caused her wrists to bend in ways they should not have.

Interestingly, when Jane started playing the alto flute at the age of sixteen, she did not experience any physical problems, possibly due to her body size and the different physical demands of the instrument. This could also be linked to her early learning experience with the flute, which required a big stretch for her small hands.

Jane faced unfairness because of the low marks received on her flute performance at the end of her undergraduate degree. The sense of unfairness led her to switch from flute performance to composition. This experience significantly impacted her, as she nearly gave up the flute. This turning point reshaped her perspective on things, and now, when she assesses others, she makes sure everything is fair, as it may impact one’s future.

The fire at Jane’s house was a traumatic event, destroying all her instruments and composition, and profoundly affecting her mentally. This occurred three months before her PhD submission, resulting in the loss of her entire PhD work. She had to start her PhD again from scratch and complete her composition without any instruments. She noted that this experience required her to develop strong resilience by accepting the loss and finding ways to learn from it. To cope with post-traumatic stress, Jane focused on small positive things, which gradually became bigger sources of positivity. She believes that accepting and learning from things beyond her control is a form of resilience.

In addition, Jane broke her elbow a few years ago and had to stop playing for a while. She sought help from a physiotherapist who told her that she would never get full use of her arm again. She questioned about this. When she started playing the flute again, she had to learn again, coming back to playing a little bit at a time, questioning everything she did. She would think, ‘Do you really need to have your hand in that position? And what if you do this?’ She used this period as an opportunity to question and revisit everything she was doing when she was playing before. During her rest and rehabilitation, she used a timer in her practice sessions to measure time, assess her condition, and decide whether to keep playing for another five minutes. Despite her ongoing right arm issues, this approach became an effective coping strategy for her. The trauma she experienced ultimately contributed to her personal growth, which she now views as an opportunity to reflect, question, and improve.

Jane reported experiencing breathing problems throughout her life and was diagnosed with asthma in 2018. She learned two distinct breathing techniques from her flute teachers, which influenced her to develop her own approach. Her method emphasises breathing naturally while controlling the speed of the movements, without the need to tense a lot of muscles unnecessarily.

Jane never had any formal training in health education but attended a few online body mapping sessions, which she finds useful. She realised that as we grow, our bodies change, and the way we play the flute has to change. This is something she has embedded into her teaching.

Overall, the outcome was that Jane managed to get back into great physical health. Jane reflects, ‘Although physical problems are seen as negative, actually for me, every time I’ve had some form of adversity, it has been one of the most positive things that could have happened once I got through it… So my approach was, I mean, it was difficult at the time, but I got through it, and I learned a lot in the process.’ Both physical and mental health issues are significant challenges for Jane. She developed strategies to manage her physical health by learning proper posture and building resilience by viewing these experiences as learning opportunities. Jane demonstrates resilience and adaptability by recovering from physical issues and rebuilding her life after a traumatic house fire. Her approach to living with these challenges is her capacity to uncover favourable results from adverse events. She demonstrates her remarkable ability to turn adversity into resources and opportunities for personal growth. Jane’s story highlights the significance of managing physical health, mental resilience, adaptability, and supportive social relationships in personal growth and professional development.

In common with other professional musicians, flautists can experience health and wellbeing challenges, resulting in physical and mental difficulties. They need resources that promote health throughout their lifespan. Each instrument places specific demands on the performer, and for flautists, the physical stance requires a particularly stressful posture. Helen and Jane exemplify how early experiences, continuous challenges, proper training, resilience, adaptability, and social support contribute to managing performance-related health and wellbeing issues. In addition, participants who are flute teachers expressed a desire for greater access to health education to enhance their knowledge of how best to promote students’ health during lessons.

## 5 Conclusion

Flautists encounter health-related challenges associated with their practice and performances. The questionnaire and interview data suggest that they each require an understanding of likely performance-related health challenges and supportive resources to assist them in managing these challenges and enhancing their overall health. Importantly, during the interviews, participants were eager to disseminate their insights into their own health education and to integrate these personal lessons into their own music pedagogy. The study highlights the importance for flute teachers in having practical knowledge of injury prevention in order for such knowledge to be a key component of their own teaching.

The participants’ experiences provide valuable insights for other flautists facing similar challenges. Although participants’ stories were diverse, certain common themes emerged, shedding light on how they manage and live with these issues. The study emphasises the importance of managing health and the value of supportive social connections. Participants cope with health challenges by integrating passion, resilience, and adaptability into their lives, finding ways to grow and continue to thrive in their flute-playing journey. In conclusion, the study underscores the need for comprehensive health education and support resources for flautists. It highlights the significance of resilience and adaptability in fostering health, wellbeing, and success in the pursuit of their musical careers. These findings highlight the potential for other music educators to benefit from the insights and experiences of these flautists, hopefully leading to more informed and holistic music instruction that prioritises health and wellbeing.

## Data Availability

The raw data supporting the conclusions of this article will be made available by the authors, without undue reservation.

## Footnotes

^1^Tendinitis and tenosynovitis: Tendinitis is inflammation of a tendon, often developing after degeneration (tendinopathy). Tenosynovitis is tendinitis with inflammation of the tendon sheath lining. Symptoms usually include pain with motion and tenderness with palpation.
